# Acrolein-Exposed Normal Human Lung Fibroblasts *in Vitro*: Cellular Senescence, Enhanced Telomere Erosion, and Degradation of Werner’s Syndrome Protein

**DOI:** 10.1289/ehp.1306911

**Published:** 2014-04-18

**Authors:** Jun-Ho Jang, Shannon Bruse, Salam Huneidi, Ronald M. Schrader, Martha M. Monick, Yong Lin, A. Brent Carter, Aloysius J. Klingelhutz, Toru Nyunoya

**Affiliations:** 1 Division of Pulmonary, Critical Care and Sleep Medicine, Department of Internal Medicine, University of New Mexico and New Mexico VA Health Care System, Albuquerque, New Mexico, USA; 2 Lovelace Respiratory Research Institute, Albuquerque, New Mexico, USA; 3 Division of Pulmonary, Critical Care, and Occupational Medicine, and; 4 Department of Microbiology, Roy J. and Lucille A. Carver College of Medicine, University of Iowa, Iowa City, Iowa, USA

## Abstract

Background: Acrolein is a ubiquitous environmental hazard to human health. Acrolein has been reported to activate the DNA damage response and induce apoptosis. However, little is known about the effects of acrolein on cellular senescence.

Objectives: We examined whether acrolein induces cellular senescence in cultured normal human lung fibroblasts (NHLF).

Methods: We cultured NHLF in the presence or absence of acrolein and determined the effects of acrolein on cell proliferative capacity, senescence-associated β-galactosidase activity, the known senescence-inducing pathways (e.g., p53, p21), and telomere length.

Results: We found that acrolein induced cellular senescence by increasing both p53 and p21. The knockdown of p53 mediated by small interfering RNA (siRNA) attenuated acrolein-induced cellular senescence. Acrolein decreased Werner’s syndrome protein (WRN), a member of the RecQ helicase family involved in DNA repair and telomere maintenance. Acrolein-induced down-regulation of WRN protein was rescued by p53 knockdown or proteasome inhibition. Finally, we found that acrolein accelerated p53-mediated telomere shortening.

Conclusions: These results suggest that acrolein induces p53-mediated cellular senescence accompanied by enhanced telomere attrition and WRN protein down-regulation.

Citation: Jang JH, Bruse S, Huneidi S, Schrader RM, Monick MM, Lin Y, Carter AB, Klingelhutz AJ, Nyunoya T. 2014. Acrolein-exposed normal human lung fibroblasts *in vitro*: cellular senescence, enhanced telomere erosion, and degradation of Werner’s syndrome protein. Environ Health Perspect 122:955–962; http://dx.doi.org/10.1289/ehp.1306911

## Introduction

Acrolein (2,3-propenal) is a reactive aldehyde used in the chemical industry for synthesis of organic compounds, in the production of methionine, and in methyl chloride refrigerant. Acrolein can be formed by incomplete combustion of gasoline, wood, plastic, tobacco products, diesel fuel, and paraffin wax and by heating cooking oil to high temperatures (Stevens and Maier 2008). Acrolein can be also generated as a product of endogenous threonine metabolism (mediated by myeloperoxidase) and/or spermine metabolism (mediated by amide oxidase) (Esterbauer et al. 1991; Faroon et al. 2008; Stevens and Maier 2008). Although acrolein is ubiquitously present in the environment, acrolein exposure through inhalation of cigarette smoke or smoke from burning of plastic or wood is generally considered to compose a large proportion of total human exposure. During inhalation of environmental tobacco smoke or cigarette smoke, the concentrations of acrolein at the airway surface can be as high as 80 μM (Eiserich et al. 1995). Acrolein rapidly penetrates cell membranes and induces oxidative stress and cytotoxicity through depletion of reductive glutathione (GSH) and inhibition of glutathione *S*-transferase (Kehrer and Biswal 2000; Li L et al. 2008; Nunoshiba and Yamamoto 1999; van Iersel et al. 1997). Acrolein (> 10 μM) can cause cell apoptosis or necrosis in various types of cultured respiratory cells, such as alveolar macrophages (Li et al. 1997), alveolar epithelial cells (Roy et al. 2009), and lung fibroblasts (Jia et al. 2009). The toxicity of acrolein is likely dependent on the cell type, redox balance, acrolein concentration, and duration of exposure.

Lung fibroblasts contact alveolar type 2 epithelial cells and underlie the distal alveoli (Borok et al. 2011). Lung fibroblasts, which might be exposed to acrolein in areas of denuded membrane following epithelial injury, have an important role in wound repair after epithelial injury (Borok et al. 2011; Rennard et al. 2006; Singh and Hall 2008).

Fibroblasts are a well-established *in vitro* model for cellular senescence (Fulcher et al. 2009; Piao et al. 2005; Schneider and Mitsui 1976). Human diploid fibroblasts cultured under normal conditions have been reported to develop an irreversible cell cycle arrest, referred to as replicative senescence (Serrano and Blasco 2001). They are susceptible to developing stress-induced premature senescence after exposure to hydrogen peroxide (Chen et al. 2004), hyperoxia (Klimova et al. 2009), or cigarette smoke (Nyunoya et al. 2006). Canonical senescence-inducing pathways include the p53 and p16-retinoblastoma protein (Rb) pathways (Campisi 2005; Satyanarayana and Rudolph 2004). The p53 pathway is activated by transcriptional induction of alternate reading frame or by activation of ataxia teleangiectasia mutated protein, both of which suppress mouse double minutes–mediated p53 degradation in the presence of DNA damage under genotoxic stresses (Di Leonardo et al. 1994; Herbig et al. 2004; Moiseeva et al. 2006; Taylor et al. 2004; Zhang et al. 2005). p53 subsequently induces cell cycle arrest through a cyclin-dependent kinase (CDK) inhibitor, p21 (Campisi 2005). p16 promotes cell cycle arrest in response to DNA damage by suppressing activity of CDKs, such as CDK4 and CDK6 (Sherr and McCormick 2002). The resultant activation of Rb (via dephosphorylation) irreversibly inhibits transcriptional activity of growth-promoting factors, such as E2F (Campisi 2005).

Acrolein is known to induce DNA interstrand cross-links (ICLs; highly toxic DNA lesions) through inhibitory effects on transcription and replication (Kozekov et al. 2003). Werner’s syndrome protein (WRN), a member of the RecQ helicase family, plays an important role in DNA ICL repair (Cheng et al. 2006). Cheng et al. (2006) reported that loss of WRN protein augments ICL-induced cell death and blocks ICL repair *in vitro.* Other *in vitro* studies found that WRN protein deficiency accelerates cellular senescence and telomere shortening (Li B et al. 2008; Szekely et al. 2005). We previously observed that acrolein reduces WRN protein in cultured fibroblasts (Nyunoya et al. 2009). However, the mechanisms of acrolein-induced down-regulation of WRN protein have not been investigated.

In the present study, we found that acrolein induced cellular senescence accompanied by activation of the p53–p21 pathway and proteasome-mediated WRN protein degradation in normal human lung fibroblasts (NHLF). siRNA (small interfering RNA)–mediated supression of p53 attenuated the effects of acrolein. Acrolein also enhanced telomere attrition. These data suggest that acrolein induces p53-mediated cellular senescence associated with telomere erosion and WRN protein instability.

## Materials and Methods

*Reagents and antibodies*. We purchased chemicals, including acrolein, from Sigma Chemical Company (St. Louis, MO) and Calbiochem (San Diego, CA); protease inhibitors from Boehringer Mannheim (Ridgefield, CT); and MG-132, a proteasome inhibitor, from Calbiochem. We obtained anti-WRN antibody from Novus Biologicals (Littleton, CO), anti-p53 and anti-p16 from Santa Cruz Biotechnology (Santa Cruz, CA), anti-hyperphospho-Rb from Cell Signaling Technology (Danvers, MA), and anti-β-actin from Sigma.

*Cell culture, cell count, and cell viability*. We cultured normal human lung fibroblasts (HFL-1 cells; ATCC, Manassas, VA) at 37°C in complete medium [Dulbecco’s modified Eagle medium (DMEM) with 10% fetal bovine serum, 1% sodium pyruvate, 1% l-glutamine, 1% (vol/vol) penicillin/streptomycin, and 25 μg/mL fungizone]. We changed the complete medium every 3 days and subcultured the cells when the cell density reached 80–90% confluence. To determine the cytotoxic effects of acrolein, HFL-1 cells were cultured in 12-well Costar tissue culture plates (Corning, Inc., Corning, NY) at a starting density of 20,000 cells/cm^2^. Twenty-four hours later, the cells were exposed to various concentrations of acrolein [0, 25, 50, or 100 μM; dissolved in sterile water (HyClone HyPure Molecular Biology Grade Water; Fisher Scientific, Pittsburgh, PA) (vehicle)] for 24 hr. Vehicle controls received sterile water at the same volume as for acrolein treatment (e.g., for the 25-μM acrolein dose, controls received 25 μL of water into 10 mL of complete medium); for comparisons with multiple concentrations of acrolein, the vehicle control received the volume of water used for the highest acrolein dose. To evaluate cell death, we stained cells with trypan blue (TB) and determined the percentage of TB-stained cells per total number of cells in the corresponding group.

For determination of cell viability, cells were plated in p100 plates (100 mm) at a starting density of 20,000 cells/cm^2^. Both medium and acrolein (0, 25, 50, and 100 μM) were refreshed every 24 hr for a total of 72 hr. The surviving cells were then redistributed into 12-well Costar tissue culture plates at a starting cell density of 50,000 cells/well and cultured in the absence of acrolein. Three days later, we determined cell viability using the TB assay and cell counts; values are expressed as the percentage of unstained cells per total number of vehicle control cells. We also monitored cell density every 3 days for up to 12 days. We performed group comparisons using one-way analysis of variance (ANOVA) with Tukey comparison or Bonferroni comparison.

*Establishment of cell lines with p53 knockdown*. We cultured HFL-1 cells in complete medium in T75 (75 cm^2^) tissue culture flasks until reaching 40% confluence. Hexadimethrine bromide (also known as polybrene), a cationic polymer, was added to the medium (final concentration of 8 μg/mL) to increase the transduction efficiency of retroviral vector (Davis et al. 2004). We then infected the cells with pBABE retroviral vector encoding siRNA targeting p53 or the control vector (a gift from C. Grandori, Fred Hutchinson Cancer Research Center) as previously described (Grandori et al. 2003; Klingelhutz et al. 1994). The cells were selected with 200 μg/mL hygromycin B for 5–7 days, and all surviving colonies were collected and pooled. Population doubling (PD) of the selected cells was calculated as 0 at this point.

*Senescence-associated* β*-galactosidase (SA* β*-gal) activity.* We performed SA β-gal staining using a modification of a previously described method (Nyunoya et al. 2006). Briefly, cell samples in 6-well culture plates were fixed with 2% formaldehyde and 0.2% glutaraldehyde in phosphate-buffered saline (PBS) for 5 min at room temperature. The plates were rinsed with PBS and incubated with an SA β-gal staining solution [40 mM sodium citrate (pH 6.0), 150 mM NaCl, 5 mM potassium ferricyanide, 5 mM potassium ferrocyanide, 2 mM magnesium chloride, and 1 mg/mL 5-bromo-4-chloro-3-indoyl β-D galactoside] for 16 hr. At a magnification of 20×, we randomly selected six fields in each well. Activity is presented as the percentage of SA β-gal–positive cells per number of total cells in each field measured in three independent experiments.

*Immunoblotting*. To determine the effects of acrolein on protein expression of p53, p21, and WRN protein, we cultured HFL-1 cells in p100 plates at a starting density of 20,000 cells/cm^2^. Both medium and 25 μM acrolein were refreshed every 24 hr for a total of 72 hr. Cells were harvested just before the first acrolein exposure (day 0 control) and every 24 hr for up to 3 days (days 1, 2, and 3). To determine the effects of p53 siRNA on p53 and WRN protein in acrolein-exposed HFL-1 cells, we cultured pBABE-transduced *p53* knockdown cells with the pBABE-hygro-sip53 vector (p53KD) or the control vector (pBH) in the presence or absence of 25 μM acrolein and harvested them 2 days after exposure.

We performed immunoblot analysis as previously described (Nyunoya et al. 2006). Briefly, each sample was normalized for all comparisons using equivalent amounts of total proteins from all adherent cells retrieved. Equal loading of the protein in each group on the blots was evaluated using anti–β-actin antibody after using Restore WB stripping buffer (Thermo Fisher Scientific, Barrington, IL). Results are expressed as the relative densitometry ratio (targeted protein/β-actin). We made group comparisons using one-way ANOVA with Tukey comparison or Bonferroni comparison.

*Real-time quantitative reverse transcription polymerase chain reaction (qRT-PCR)*. We prepared RNA samples using the RNeasy mini kit (Qiagen,Valencia, CA). We performed RT-RCR and quantitative analysis for *WRN* and *GAPDH* mRNA using Taqman One-Step RT-PCR Master Mix Reagents (Applied Biosystems, Carlsbad, CA) with the following probes: *WRN* (Hs00172155_m1) and *GAPDH* (Hs99999905_m1), both from Applied Biosystems (Contreras et al. 2013).

*Chemical proteosome inhibition*. HFL-1 cells were cultured in the absence (sterile water vehicle control) or presence of 25 μM acrolein for 48 hr and then incubated with 10 μM MG-132 or vehicle (DMSO) for 2 hr.

*Acrolein exposure and determination of cell PD*. We determined PD times as previously described by Klimova et al. (2009). To determine the cell PD and telomere length (TL), we seeded cells at a starting density of 0.4 × 10^6^ cells/75 cm^2^ flask 24 hr prior to treatments and treated the cells with 10 μM acrolein. We changed the medium every 3 days (with or without adding acrolein) until the cells reached 80–90% confluence. Cells were then redistributed at the same starting density, and a subset of cells was collected for measurement of TL. Cells were considered senescent, and the experiment was terminated when the cell density failed to reach the starting density for 2 weeks. We performed cell counts using an electric particle counter (Beckman Coulter Electronics, Indianapolis, IN). PD was calculated with the formula log_2_ (*n*/0.4 × 10^6^), where *n* is the number of cells counted at subculture).

*TL (quantitative PCR)*. We prepared DNA samples using a QIAamp DNA blood kit (Qiagen, Valencia, CA) and measured relative TL using a modified quantitative PCR protocol as previously described (Cawthon 2002). Primers were obtained from Integrated DNA Technologies (Coralville, IA), The primer sequences were as follows: Tel 1: 5´-GGTT​TTTG​AGGG​TGAG​GGTG​AGGG​TGAG​GGT-3´; Tel 2: 5´-TCCC​GACT​ATCC​CTAT​CCCT​ATCC​CTAT​CCCT​ATCC​CTA-3´; 36B4u: 5´-CAGC​AAGT​GGGA​AGGT​GTAA​TCC-3´; 36B4d: 5´-CCCA​TTCT​ATCA​TCAA​CGGG​TACA​A-3´. The reference single copy gene *36B4* was used as a loading control. The final concentrations of the primers (Integrated DNA Technologies, Coralville, IA) were 270 nM for tel 1, 900 nM for tel 2, 270 nM for 36B4u, and 900 nM for 36B4d. The DNA samples were assayed in triplicate on a PCR plate. Each well contained 12.5 μL SYBR Green PCR Master Mix (Applied Biosystems) and a final DNA concentration of 1.44 μg/μL. The reactions were performed in an ABI PRISM 7900 HT Sequence Detection System (Applied Biosystems). The thermal cycling profile was as follows: stage 1, 30 min at 48°C; stage 2, 10 min at 95°C; and stage 3, 15 sec at 95°C and 1 min at 60°C (40 cycles). Relative TL was quantified as the telomere/single copy gene *36B4* (T/S) ratio, calculated according to Cawthon’s formula (Cawthon 2002).

*Effects of acrolein on telomere shortening*. We used PCR to measure relative TL at PD upon replicative senescence (46 PD) and acrolein-induced senescence (30 PD) in HFL-1 cells and the equivalent PD (30 PD) in vehicle (sterile water) control HFL-1 cells. We determined the relative TL at the PD upon acrolein-induced cellular senescence in pBH cells (10 PD) and the equivalent PD in vehicle control pBH cells and in both unexposed and exposed p53KD cells. In addition, we evaluated relative TL at the PD upon replicative senescence (41 PD) and upon acrolein-induced senescence (31 PD) in p53KD cells, as well as the equivalent PD in vehicle control p53KD cells. We made group comparisons using one-way ANOVA with Tukey comparison. Relative TL is expressed as mean ± SEM of triplicate samples.

*Statistical analysis*. We made group comparisons using one-way ANOVA with Tukey comparison (if all pairwise differences were of interest), or with Bonferroni comparison (if only a few pairwise differences were of interest) to control familywise error rate from multiple comparisons. Data are expressed as mean ± SEM, and *p* < 0.05 is considered statistically significant.

## Results

**Figure 1:**
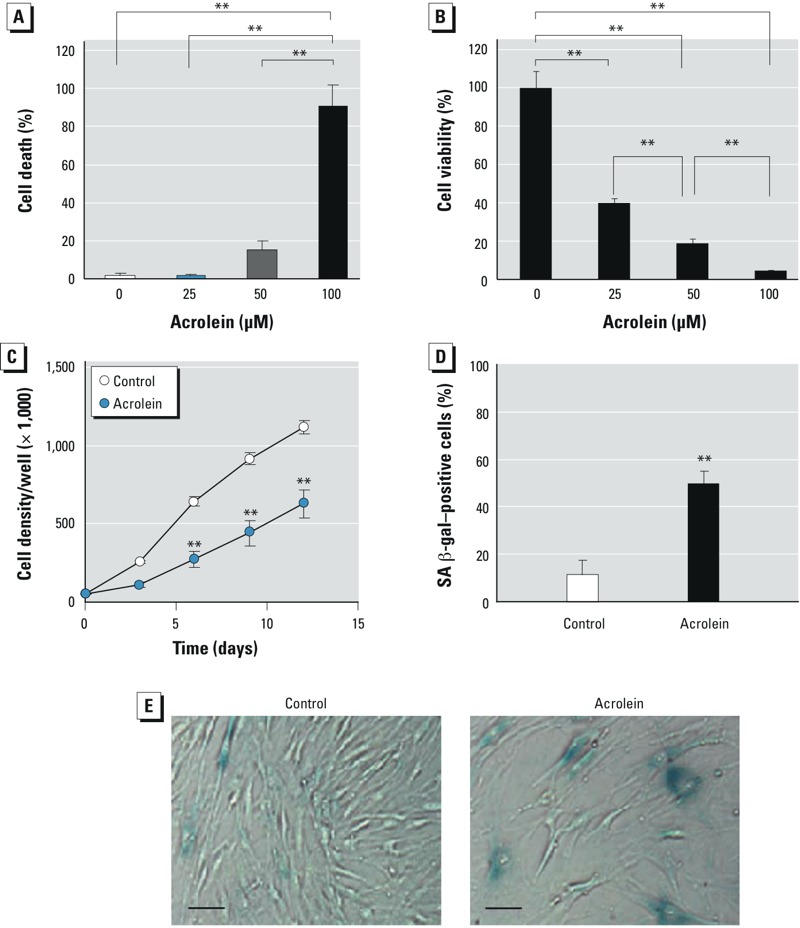
Effects of acrolein on cellular senescence. Cell death (*A*) and cell viability (*B*) of HFL‑1 cells cultured in the absence (vehicle control; 0 μM) or presence of acrolein (25, 50, or 100 μM) for 24 hr and 3 days, respectively. Values for cell death represent the percentage of TB‑stained cells in the total number of cells in the corresponding group, and values for cell viability represent the percentage of unstained cells in the total number of control cells. (*C*) Cell density of HFL‑1 cells that were cultured in the absence (control) or presence of 25 μM acrolein for 3 days, with surviving cells then subcultured at a starting density of 50,000/well. Cell density was monitored every 3 days for up to 12 days. (*D,E*) SA β-gal activity in HFL‑1 cells treated as in *C* and evaluated 3 days after subculture. (*D*) Percentage of SA β-gal–positive cells per total cell number 3 days after subculture and (*E*) representative photomicrographs (bars = 5 μm). In *A–D*, data are expressed as mean ± SEM of three independent experiments. **p < 0.01.

*Effects of acrolein on cellular senescence in NHLF*. To examine whether acrolein induces cellular senescence in cultured NHLF, we first determined the cytotoxic concentration of acrolein. In HFL-1 cells cultured with acrolein [0 (vehicle control), 25, 50, and 100 μM] for 24 hr, we observed a trend toward significant cell death in the 50-μM acrolein group [*p* = 0.08 compared with vehicle control cells (0 μM acrolein)] and robust cytotxicity in the 100-μM acrolein group (Figure 1A). We then cultured HFL-1 cells with or without various concentrations of acrolein for 3 days, with the culture medium and acrolein refreshed every 24 hr. The effects of repetitive acrolein exposure (three times) on cell viability were determined by cell counts and the TB assay at 72 hr. Repetitive acrolein exposure reduced cell viability in a dose-dependent manner (Figure 1B).

Cells that survived repetitive exposure to acrolein (25 μM) were subcultured in complete medium in the absence of acrolein at a starting density of 50,000 cell/well and monitored for cell growth every 3 days for a total of 12 days. During that time period, cell growth was significantly reduced in cells previously exposed to acrolein (Figure 1C). To further characterize acrolein-induced growth inhibition, we measured SA β-gal activity in cells 3 days after subculture. Cells previously exposed to acrolein exhibited a significant increase in SA β-gal activity compared with control cells (Figure 1D,E). These data suggest that acrolein is sufficient to induce cellular senescence in cultured lung fibroblasts. A lower concentration of acrolein (10 μM) (3-day exposure) did not inhibit cell growth (see Supplemental Material, Figure S1) or increase SA β-gal activity (data not shown).

**Figure 2:**
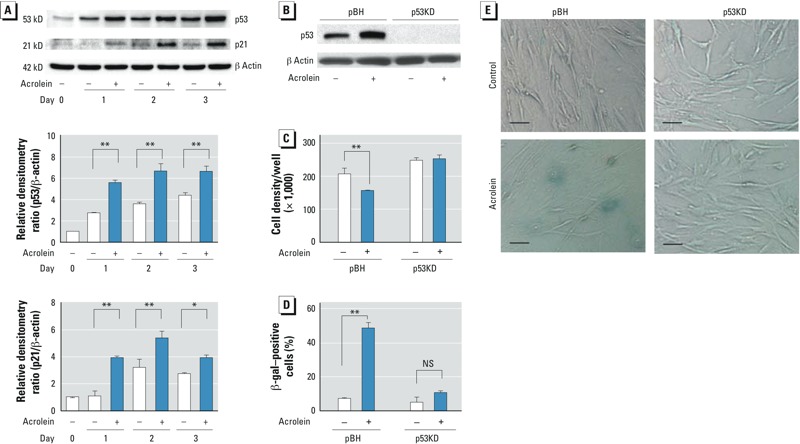
Effects of siRNA-mediated p53 suppression on cellular senescence. (*A*) p53 and p21 proteins in HFL‑1 cells cultured in the absence (–) or presence (+) of 25 μM acrolein for 1, 2, or 3 days. Immunoblot analysis of p53 and p21 (top), and the relative densitometry ratio for p53/β‑actin (center) and p21/β‑actin (bottom); each day 0 control was regarded as 1.0. (*B*) Immunoblot analysis of transduced HFL‑1 cells [p53KD (p53-deficient) and pBH (control)] cultured in the presence or absence of 25 μM acrolein for 2 days; the blot is representative of three experiments. (*C–E*) p53KD and pBH cells were cultured for 3 days with or without 25 μM acrolein and then subcultured (at a starting density of 50,000 cells/well) for 3 days. (*C*) Cell density of subcultured p53KD and pBH cells. (*D*) Percentage of SA β-gal–positive cells per total cell number 3 days after subculture. (*E*) Representative photomicrographs of p53KD and pBH cells 3 days after subculture (bars = 5 μm. In *A, C, *and *D*, data are expressed as mean ± SEM of three independent experiments. NS, not significant. *p < 0.05. **p < 0.01.

*Effects of siRNA-mediated p53 suppression on cellular senescence in NHLF*. We cultured HFL-1 cells in the presence or absence of 25 μM acrolein for up to 3 days to determine whether acrolein activates the canonical senescence-inducing pathways p53–p21 and p16-Rb (Campisi 2005). Although both p53 and p21 levels were higher in acrolein-exposed cells than in controls during the period, they appeared to be slightly lower on day 3. In addition, both p53 and p21 levels did increase in control cells over the 3-day period (Figure 2A). In contrast, acrolein did not alter p16 expression (see Supplemental Material, Figure S2). These data suggest that acrolein induces cellular senescence accompanied by activation of the p53–p21 pathway.

To determine whether p53 is required for acrolein-induced cellular senescence, we established p53-deficient fibroblasts by stable transduction using a retroviral vector encoding siRNA (p53KD), which targets p53, as shown in Figure 2B. We cultured these fibroblasts in the presence or absence of 25 μM acrolein for 3 days, and harvested cells for immunoblot analysis at 2 days. Suppression of p53 prevented acrolein-induced growth inhibition (Figure 2C). Consistent with the cell growth data, p53 knockdown significantly attenuated SA β-gal activity in acrolein-exposed cells (Figure 2D,E). These data suggest that acrolein induces cellular senescence via p53 activation in cultured lung fibroblasts.

**Figure 3:**
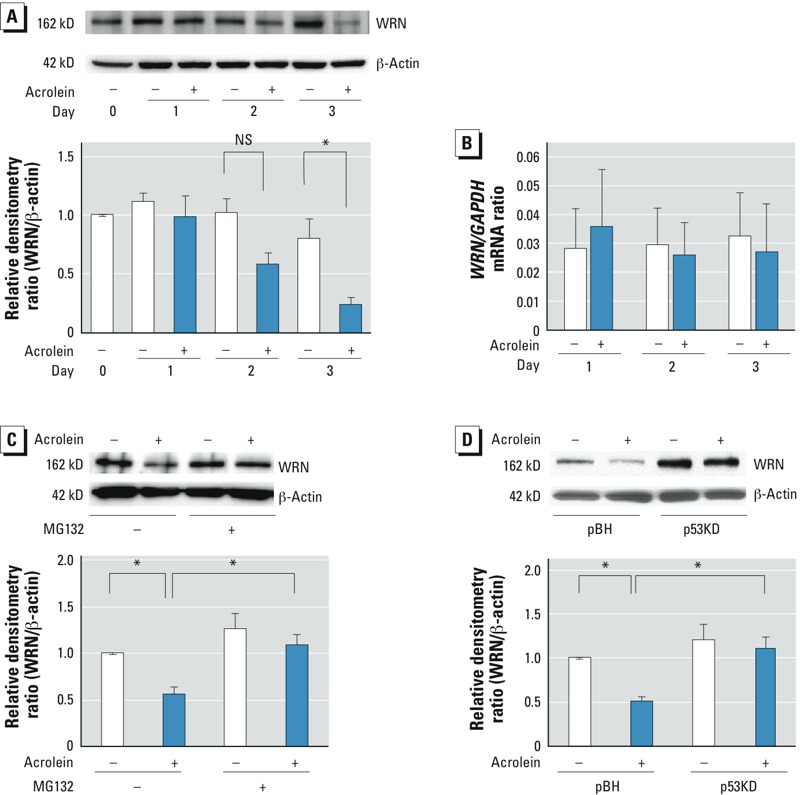
Effects of proteasome inhibition on acrolein-induced WRN protein degradation. WRN protein (*A*) and *WRN* and *GAPDH* mRNA (*B*) in HFL‑1 cells cultured in the absence (–) or presence (+) of 25 μM acrolein for 1, 2, or 3 days. (*A*) Protein levels were evaluated by immunoblot analysis (top); values are expressed as the relative densitometry ratio (WRN protein/β-actin), with each day 0 control regarded as 1.0 (bottom). (*B*) Steady-state levels of *WRN* and *GAPDH* mRNA were quantified by RT-PCR; values are expressed as the ratio of *WRN* to the *GAPDH* control. (*C*) WRN protein in HFL‑1 cells cultured without or with 25 μM acrolein for 48 hr and then incubated with 10 μM MG-132 or DMSO vehicle for 2 hr. (Top) Protein levels were evaluated by immunoblot analysis; (bottom) values are expressed as the relative densitometry ratio (WRN protein/β-actin), with the vehicle control regarded as 1.0. (*D*) WRN protein in transduced HFL‑1 cells [p53KD (p53-deficient) and pBH (control)] [p53KD (p53-deficient) or pBH (control)] cultured in the absence or presence of 25 μM acrolein for 2 days. (Top) Protein levels were evaluated by immunoblot analysis; (bottom) values are expressed as the relative densitometry ratio (WRN protein/ β-actin), with the pBH control regarded as 1.0. For *A–D*, data are expressed as mean ± SEM of three independent experiments. NS, not significant. *p < 0.05.

*Effects of proteasome inhibition on acrolein-induced WRN protein degradation*. Acrolein forms DNA ICLs (Kozekov et al. 2003) that sensitize WRN-deficient cells to cytotoxicity (Cheng et al. 2006). In HFL-1 cells exposed to 25 μM acrolein, steady-state levels of WRN were reduced at 48 hr (*p* = 0.07) and significantly reduced at 72 hr (*p* = 0.01) (Figure 3A). To determine the level at which WRN protein expression was regulated, we quantified the steady-state levels of *WRN* mRNA by RT-PCR in acrolein-exposed or control fibroblasts. Acrolein did not alter steady-state levels of *WRN* mRNA at any time point after exposure (Figure 3B). To determine whether acrolein-induced WRN protein down-regulation is mediated by proteasome-dependent degradation, we cultured HFL-1 cells in the presence or absence of 25 μM acrolein for 48 hr and then incubated the cells with 10 μM MG-132, a proteasome inhibitor, for 2 hr. MG-132 attenuated acrolein-induced WRN protein degradation (Figure 3C). These data suggest that acrolein enhances proteasome-dependent WRN protein degradation.

We then examined the effects of siRNA-mediated knockdown of *p53* on WRN protein expression in acrolein-exposed lung fibroblasts by culturing p53-deficient HFL-1 cells in the presence or absence of acrolein for 48 hr. Steady-state levels of WRN protein were determined by immunoblot analysis. p53 knockdown appeared to increase WRN protein expression compared with the control and to prevent acrolein-induced WRN protein down-regulation (Figure 3D). Data suggest that p53 is necessary for acrolein-induced WRN protein down-regulation.

**Figure 4:**
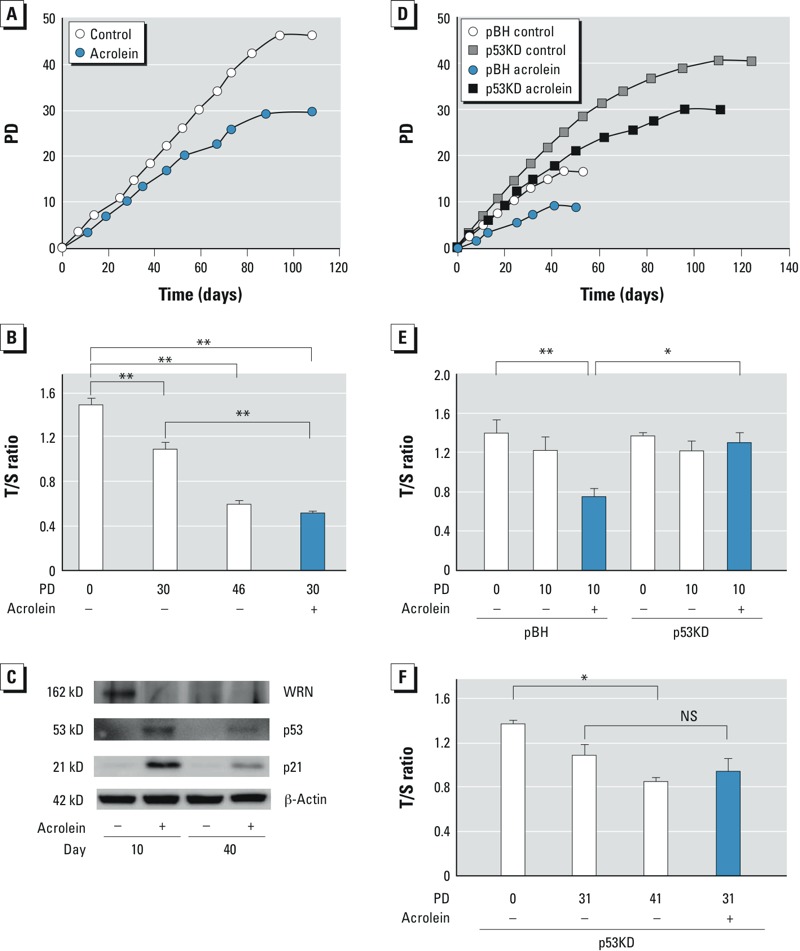
Effects of acrolein on population doubling (PD) and telomere length (TL). PD (*A*) and TL (*B*) in HFL‑1 cells cultured in the absence (–) or presence (+) of 10 μM acrolein. Relative TL is expressed as the mean ± SEM of triplicate samples at the PD upon acrolein-induced senescence (30 PD), the equivalent PD (30 PD) in control cells, and replicative senescence (46 PD) in control cells. (*C*) Immunoblot analysis of p53, p21, and WRN protein in HFL‑1 cells at day 10 and day 40 of culture in the absence (–) or presence (+) of 10 μM acrolein. PD (*D*) and and TL (*E*) in p53KD (p53-deficient) or pBH (control) cells cultured in the absence (–) or presence (+) of 10 μM acrolein. The relative TL is expressed as mean ± SEM of triplicate samples at the PD upon acrolein-induced cellular senescence in pBH cells (10 PD), and the equivalent PD in unexposed pBH cells and p53KD cells in the presence or absence of acrolein. (*F*) TL in p53KD cells cultured in the absence or presence of 10 μM acrolein. The relative TL is expressed as the mean ± SEM T/S ratio of triplicate samples at the PD upon replicative senescence (41 PD) and acrolein-induced senescence (31 PD) in p53KD cells, and the equivalent PD in unexposed p53KD cells. NS, not significant. *p < 0.05. **p < 0.01.

*Effects of acrolein on telomere shortening in NHLF*. To determine the effects of acrolein on TL, we cultured HFL-1 cells in the presence or absence of acrolein, and monitored the PD. We initially used 25 μM acrolein for this experiment based on the cytotoxicity data (Figure 1A), but HFL-1 cells did not survive the continuous exposure to 25 μM acrolein > 7 days (100% cell death). We thus reduced the concentration to 10 μM acrolein for the prolonged-exposure experiments. At the end of the time course, the PD was 35% lower for acrolein-exposed cells than for control cells (Figure 4A). We measured the TL for acrolein-exposed fibroblasts at the end point of 30 PD (acrolein-induced senescence) and the control fibroblasts at three time points: the starting point (0 PD), 30 PD, and the end point (46 PD). As we expected, TL was reduced in control fibroblasts with higher PD (Figure 4B). In acrolein-exposed cells at 30 PD, TL was significantly reduced compared with controls at 30 PD. In fact, the TL in acrolein-treated cells at 30 PD was similar to that in control cells at 46 PD, when normal replicative senescence occurred. These data suggest that acrolein accelerates telomere attrition in lung fibroblasts.

We examined expression of p53, p21, and WRN proteins at days 10 and 40 (Figure 4C). Acrolein-exposed cells showed increases in both p53 and p21 but a decrease in WRN at day 10. The effects of acrolein were reduced at day 40. WRN protein expression was also reduced with advanced PDs at day 40 in control cells. These data suggest that the effects of acrolein on p53 and p21 induction and WRN protein down-regulation are prominent at the early time point but are attenuated over the prolonged period.

To determine the effects of p53 suppression on acrolein-induced growth inhibition and telomere shortening, we performed the PD assay using p53KD cells (p53 knockdown) and pBH cells in the presence or absence of 10 μM acrolein. In vehicle controls, those with suppressed p53 had a PD of 41, whereas pBH cells had a PD of 18 (Figure 4D). Although the PD was decreased in both p53KD cells and pBH cells treated with acrolein, p53KD cells were more resistant to acrolein-induced growth inhibition than were pBH cells (p53KD cells, 24% reduction, pBH cells, 44% reduction, compared with corresponding control cells). As we expected, acrolein-treated pBH cells had significantly accelerated telomere shortening compared with untreated cells (Figure 4E). In contrast, suppression of p53 ameliorated the effects of acrolein on telomere shortening. Further culturing of the p53KD cells showed no significant difference in TL between untreated and acrolein-treated cells at 31 PD (Figure 4F). This suggests that p53 is required for acrolein-induced telomere erosion.

## Discussion

In the present study, we found that acrolein induced cellular senescence characterized by growth inhibition and an increase in SA β-gal activity *in vitro*. Acrolein induced expression of both p53 and p21. *p53* knockdown prevented cellular senescence, WRN protein degradation, and enhanced telomere attrition in acrolein-exposed cells. These results suggest that acrolein induces p53-mediated cellular senescence accompanied by enhanced WRN protein instability and telomere shortening.

Although little is known about the effects of acrolein on cellular senescence, several *in vitro* studies have examined the effects of acrolein on cytotoxicity (Jia et al. 2009; Li et al. 1997; Roy et al. 2009). Those studies found that acrolein increased cytotoxicity in a dose-dependent manner; the predominant type of cell death (approximately 95%) was apoptosis in acrolein-exposed A549 cells (an immortalized alveolar epithelial cell line) (Roy et al. 2010). Of note, acrolein-induced cytotoxicity, associated with a reduction of glutathion, was rescued by R-α-lipoic acid (a thiol antioxidant) (Jia et al. 2009). Other *in vitro* studies found that thiols, especially glutathion, are important for cell survival against acrolein (Dypbukt et al. 1993; Krokan et al. 1985). These findings support the idea that oxidative stress plays a crucial role in acrolein-induced cytotoxicity. In fact, oxidative stress by hydrogen peroxide or hyperoxia can also accelerate cellular senescence in cultured fibroblasts (Chen et al. 2004; von Zglinicki et al. 1995). Oxidative stress induced by acrolein may contribute to premature senescence; in future studies, it will be of interest to examine the potential mechanism by which this occurs. It should also be noted that the dose of acrolein used may lead to different outcomes. In the present study, we used a much lower concentration of acrolein (10–25 μM) than did Jia et al. (2009) in their *in vitro* study using NHLF (100 μM). We speculate that prolonged and repetitive exposure to relatively low concentrations of acrolein more effectively induces cellular senescence rather than apoptosis.

Telomeres consist of repetitive nucleotide sequences (TTAGGG)n located at chromosomal ends. A critically short TL is sufficient to induce cellular senescence through activation of the DNA damage response (Maser and DePinho 2004). To our knowledge, no previous study has determined the effects of acrolein on telomere length *in vitro*. In the present study, we found that prolonged acrolein exposure enhanced telomere shortening compared with control fibroblasts. This could be due to the effects of acrolein on DNA damage, given that acrolein forms DNA adducts of guanosine nucleotides, which are enriched in telomeres. Oxidative stress may be directly involved in DNA damage to telomeres (Jia et al. 2009). Prolonged exposure to a mildly elevated concentration of oxygen (40%) also enhances telomere shortening in NHLF (von Zglinicki et al. 1995).

p53 is one of most extensively studied tumor-suppressor proteins and plays an important role in cell fate, including transient cell cycle arrest, cellular senescence, or cell death under genotoxic stress (Rufini et al. 2013). Roy et al. (2010) recently reported that acrolein induced apoptosis via p53 activation and that acrolein-induced apoptosis was attenuated by pretreatment with either pifithrin-α, a chemical inhibitor of p53, or polyethylene glycol–conjugated catalase in A549 cells. We found that acrolein also activated p53 in NHLF. This p53 activation was necessary for acrolein-induced cellular senescence. By contrast, p16, another canonical senescence-inducing pathway (Krishnamurthy et al. 2004) was not increased in acrolein-exposed fibroblasts. Although no study has determined the effects of acrolein on p16 expression, hydrogen peroxide has been reported to increase p16 protein expression in addition to activating the p53–p21 pathway in NHLF (Chen et al. 2004). These results suggest that the type of DNA-damaging agent determines a specific senescence-inducing pathway in NHLF. In a previous study, we reported that cigarette smoke extract induced fibroblast senescence accompanied by activation of both p53/p21 and p16 pathways (Nyunoya et al. 2006). Because cigarette smoke contains numerous toxic chemicals and reactive oxygen/nitrogen species and > 60 other carcinogens (Cerami et al. 1997; Church and Pryor 1985; Pryor and Stone 1993; Smith et al. 2003), it is likely that the other toxic chemicals induce p16 activation. In fact, Zhou et al. (2003) reported that 4-(methylnitrosamino)-1-(3-pyridyl)-1-butanone (NNK), a known tobacco-speciﬁc nitrosamine and carcinogen, induced p16 in immortalized airway epithelial cells.

WRN protein plays a crucial role in repairing DNA ICLs generated by acrolein (Cheng et al. 2006; Kozekov et al. 2003). In the present study, we found that acrolein augmented proteasome-dependent and p53-mediated WRN protein degradation. WRN protein is constitutively expressed and regulated by the Sp1 transcription factor that is suppressed by p53 (Yamabe et al. 1998). However, we observed that *WRN* mRNA levels were not reduced, despite p53 activation. These data suggest a novel role for p53 to decrease WRN protein stability in a proteasome-dependent manner. This potential mechanism is not without precedent in that Kaluzová et al. (2004) observed that p53 activation through DNA damage enhances proteaosome-dependent degradation of hypoxia inducible factor-1 in hypoxic cells.

Our current results suggest that acrolein is a critical component of cigarette smoke–induced WRN protein degradation. Wang et al. (2012) showed that acrolein inhibited multiple DNA repair pathways in both normal human bronchial epithelial cells and NHLF. Further, consistent with our findings of WRN protein degradation, acrolein enhanced proteasome-dependent degradation of the key factors of DNA repair, including *XPA*, *XPC*, *OGG1*, *MLH1*, and *PMS2* (Wang et al. 2012). These results suggest that acrolein inhibits multiple pathways of DNA repair leading to mutagenesis. In particular, acrolein-induced WRN protein degradation may contribute to cellular senescence because WRN protein loss markedly reduces proliferative capacity *in vitro* (Li B et al. 2008).

Emerging evidence suggests a potential role of accelerated aging in cigarette smoke–induced emphysema (Aoshiba and Nagai 2009; Ito and Barnes 2009). Holz et al. (2004) observed that lung fibroblasts isolated from smokers with emphysema exhibited a senescent phenotype compared with smokers without emphysema. Given the abundance of acrolein in cigarette smoke and the relatively long half-life (Ghilarducci and Tjeerdema 1995), acrolein may play a crucial role in the development of smoking-induced emphysema.

The limitation of this study is that we examined the effects of acrolein on cellular senescence using only one cell type (lung fibroblasts). Obviously, alveolar macrophages and airway and alveolar epithelial cells are also primary targets of acrolein. However, it would be difficult to study cellular senescence using these cell types. For example, alveolar macrophages are usually terminally differentiated and thus cannot proliferate. Primary human bronchial epithelial cells have a much lower PD (~ 10) compared with NHLF (~ 50) (Fulcher et al. 2009; Piao et al. 2005; Schneider and Mitsui 1976). Furthermore, little is known about the *in vivo* effects of acrolein on cellular senescence, although acrolein exposure induced emphysema accompanied by CD8^+^ T-cell–mediated lung inflammation and both airway and alveolar epithelial cell apoptosis in a mouse model (Borchers et al. 2007).

In the present study, we observed that acrolein induced p53-mediated cellular senescence accompanied by accelerated telomere shortening and enhanced WRN protein degradation *in vitro*. These results suggest a potential role of acrolein in accelerated aging through inhibition of DNA repair.

## Supplemental Material

(639 KB) PDF
