# Supplementary material for: Acrolein-Exposed Normal Human Lung Fibroblasts in Vitro: Cellular Senescence, Enhanced Telomere Erosion, and Degradation of Werner’s Syndrome Protein
Source: Environ Health Perspect. 2014 Apr 18;122(9):955–62. doi: 10.1289/ehp.1306911 (PMC4154210; doi:10.1289/ehp.1306911)
Supplement: (639 KB) PDF [file ehp.1306911.s001.pdf]

Environmental Health Perspectives doi:10.1289/ehp.1306911

Supplemental Material

Acrolein-Exposed Normal Human Lung Fibroblasts *in Vitro*: Cellular Senescence, Enhanced Telomere Erosion, and Degradation of Werner's Syndrome Protein

Jun-Ho Jang, Shannon Bruse, Salam Huneidi, Ronald M. Schrader, Martha M. Monick, Yong Lin, A. Brent Carter, Aloysius J. Klingelhutz, and Toru Nyunoya

Figure S1

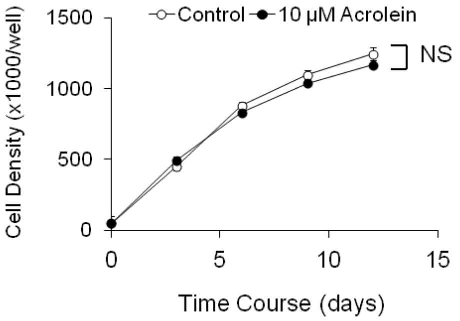

Figure S1. Effects of a short-term exposure to acrolein on cell growth in cultured normal human lung fibroblasts. HFL-1 cells were cultured in the absence or presence of 10 μM acrolein for 3 d. The cells were subcultured at a starting density of 50,000/well. The cell density was monitored every 3 d for up to 12 d. Data are expressed as mean ± SEM for three independent experiments.

Figure S2

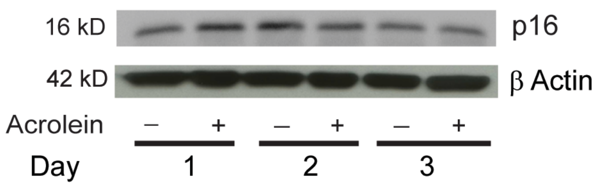

Figure S2. Effects of acrolein on p16 protein expression in cultured normal human lung fibroblasts. HFL-1 cells were cultured with or without 25 μM acrolein for various periods (1, 2, and 3 d). Immunoblot analysis was performed for p16. Equal loading was determined by stripping the blot and reprobing with antibodies to β-actin. Immunoblotting data are representative of three experiments.
